# Deposition of respiratory virus pathogens on frequently touched surfaces at airports

**DOI:** 10.1186/s12879-018-3150-5

**Published:** 2018-08-29

**Authors:** Niina Ikonen, Carita Savolainen-Kopra, Joanne E. Enstone, Ilpo Kulmala, Pertti Pasanen, Anniina Salmela, Satu Salo, Jonathan S. Nguyen-Van-Tam, Petri Ruutu, for the PANDHUB consortium, Nadezhda Gotcheva, Raija Koivisto, Anna-Maria Veijalainen, Nicolas Poirot, Nabila Laajail, Emma Bennett, Ian Hall, Stephane Bastier, Yann Lapeyre, Audrey Berthier

**Affiliations:** 1 Department of Health Security, National Institute for Health and Welfare, P.O.Box 30, 00271 Helsinki, Finland; 2 School of Medicine, Division of Epidemiology and Public Health, University of Nottingham, Nottingham, UK; 3 VTT Technical Research Centre of Finland Ltd, Espoo and Tampere, Finland; 4 Department of Environmental and Biological Sciences, University of Eastern Finland, Kuopio, Finland

**Keywords:** Influenza virus, Respiratory virus, Surface contamination, Airport

## Abstract

**Background:**

International and national travelling has made the rapid spread of infectious diseases possible. Little information is available on the role of major traffic hubs, such as airports, in the transmission of respiratory infections, including seasonal influenza and a pandemic threat. We investigated the presence of respiratory viruses in the passenger environment of a major airport in order to identify risk points and guide measures to minimize transmission.

**Methods:**

Surface and air samples were collected weekly at three different time points during the peak period of seasonal influenza in 2015–16 in Finland. Swabs from surface samples, and air samples were tested by real-time PCR for influenza A and B viruses, respiratory syncytial virus, adenovirus, rhinovirus and coronaviruses (229E, HKU1, NL63 and OC43).

**Results:**

Nucleic acid of at least one respiratory virus was detected in 9 out of 90 (10%) surface samples, including: a plastic toy dog in the children’s playground (2/3 swabs, 67%); hand-carried luggage trays at the security check area (4/8, 50%); the buttons of the payment terminal at the pharmacy (1/2, 50%); the handrails of stairs (1/7, 14%); and the passenger side desk and divider glass at a passport control point (1/3, 33%). Among the 10 respiratory virus findings at various sites, the viruses identified were: rhinovirus (4/10, 40%, from surfaces); coronavirus (3/10, 30%, from surfaces); adenovirus (2/10, 20%, 1 air sample, 1 surface sample); influenza A (1/10, 10%, surface sample).

**Conclusions:**

Detection of pathogen viral nucleic acids indicates respiratory viral surface contamination at multiple sites associated with high touch rates, and suggests a potential risk in the identified airport sites. Of the surfaces tested, plastic security screening trays appeared to pose the highest potential risk, and handling these is almost inevitable for all embarking passengers.

## Background

The continuous growth in air travel [1] increases the likelihood of rapid spread of infectious diseases between countries and continents. Air travel made possible the rapid spread of Severe Acute Respiratory Syndrome (SARS) from Hong Kong in 2003 to several countries in a very short time [2], as was the case for the global spread of pandemic influenza A(H1N1)pdm09 from Mexico and the United States of America in 2009 [3].

Symptomatic and asymptomatic respiratory tract infections are common among passengers [4], with potential for transmission to fellow passengers during pre-embarkation and travel, or after arrival at destination, via multiple modes of transmission, including airborne, droplet and contact transmission. Transmission of a range of infections during air travel has been investigated and recommendations for control and incident investigation have been published [5–9]. Confirmed influenza transmission has also been reported aboard ships [10], and transmission of influenza-like illness has been reported aboard ships [11] and trains [12]. The potential for airports to spread an infection causing pandemic threat globally has been modelled estimating how individual airports could contribute to an epidemic process [13].

Major traffic hubs, particularly large airports receive passengers from multiple continents [14, 15]. There is little published literature on the role of airports or other major hubs (e.g. ports and railway stations) in the transmission of infections, or on the main risk points within a hub for transmission. One published event involved a patient travelling through an airport with measles (which transmits efficiently through air in closed premises), where epidemiological investigation showed transmission to other passengers in the airport [16].

Virus sampling of the touched environment has been previously performed in many settings, including for example, hospitals, homes of patients infected with influenza [17, 18], children’s nurseries [19], homes of people infected with rhinovirus [20] and a hotel setting [21]. To our knowledge, only one such study has been published pertaining to an airport environment, which found that out of 40 surfaces tested, 17.5% were positive for at least one of a number of viral pathogens, including influenza. [22]. We have supplemented these findings by investigating the presence of respiratory viruses in the passenger environment of an airport in order to identify risk points and guide measures to minimize transmission.

## Methods

### Study site and sampling

Helsinki-Vantaa airport is the main airport in Finland, with a throughput of 18.9 million passengers in 2017. Approximately 12% of the traffic is to or from Eastern, South-Eastern and Southern Asia.

**Fig. 1:**
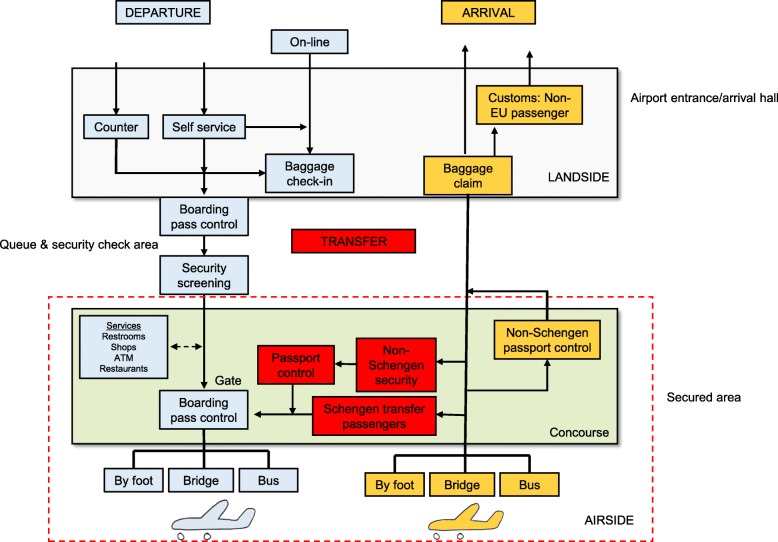
Passenger processes in the Helsinki – Vantaa Airport

The passenger processes within the airport of departing, transit and arriving passengers were carefully mapped during an initial site visit, going through the actual passenger pathway with hub staff, to identify surfaces which are frequently touched, and areas where passenger density would be high (where direct transmission of respiratory viral pathogens could potentially take place) (Fig. 1). After a pilot phase in September, 2015, to test sampling procedures, sampling for the study was performed in February 2016 at the peak period of the 2015–16 annual influenza epidemic in Finland [23].

Surface and air samples were collected weekly at three different time points (weeks 5–7/2016: 4.2.2016–17.2.2016) from a variety of sites along the passenger flow pathways in Helsinki-Vantaa airport (Table 1) from frequently touched surfaces. The hours of sampling were immediately after the early morning peak traffic (0700- 0900 h), after the noon peak (1100-1200 h), and after the mid-afternoon peak hours (1400 h – 1600 h), during which much of the transit traffic takes place for passengers travelling between Central European and Asian destinations. Sampling time was tailored so that the surfaces sampled had not been cleaned after the most recent preceding traffic peak.

**Table 1:** Respiratory viruses detected from the surface and air samples

Sample type	Sampling area	Positive/number of samples	Detected respiratory virus
Surface	Toilet: upper surface the toilet bowl lid	0/14	none
Surface	Toilet: button for flushing	0/14	none
Surface	Toilet: lock at the door inside the toilet	0/14	none
Surface	Hand-carried luggage boxes at the security check area	4/8	adeno
			influenza A
			rhino
			human corona OC43
Surface	Armrest of a chair at the waiting area	0/6	none
Surface	Handrails of an escalator	0/10	none
Surface	Handrails of stairs	1/7	human corona OC43
Surface	Plastic toy dog in children’s playgroung	2/3	rhino
			adeno
Surface	The trolley handles for luggage	0/3	none
Surface	The buttons of an elevator	0/3	none
Surface	The touch screen on the check-in machine	0/3	none
Surface	Desk and divider glass at the passport control point	1/3	rhino
Surface	Buttons of payment terminal at the pharmacy	1/2	rhino and human corona OC43
Air	At the security check area	1/4	adeno

The surface samples were taken using nylon swabs, which were immersed in viral transport medium (VTM) before sampling. The standard sampling area size was 10 × 10 cm (swab applied in horizontal followed by vertical and diagonal sweeps). For security screening trays at the security check area, the sample was taken from all the outer sides of the tray using the same swab, moving it horizontally, vertically and transversely across the sampling area, including the area just below the tray’s lip. For the toilet door knobs and flushing buttons the swabbing covered the entire touchable surface. The swab was immediately placed into 1 ml of VTM.

Four air samples were taken during the study period, two samples at two different times of the day in week 5 and one sample in weeks 6 and 7. The air particles were collected using an Impactor FH5® sampler (Klotz GmbH, Germany) with filtration [24]. The sampler was positioned at approximately 2 m from the floor in the passenger security check area and ran for 33 min. The flow rate through the sampler was 30 L/min giving a total sample volume of 1000 L filtered through a gelatin filter paper (Gelatin Filter Disposables, Sartorius Stedim Biotech GmbH, Germany). Before nucleic acid extraction, an approximately 0.5 × 5 cm strip of the gelatin filter paper was immersed and dissolved in 1 ml VTM.

All samples were transported refrigerated and stored in refrigerator at approximately + 4 °C for short-term storage (maximum 24 h), and then frozen at approximately − 60 °C for extended storage before nucleic acid extraction and virus detection.

### Nucleic acid extraction and virus detection

Viral nucleic acid was extracted from 100 μl samples with the Qiagen Qiacube® instrument using RNeasy Mini Kit® (Qiagen, Hilden, Germany) following the manufacturer’s instructions and was eluted in 50 μl. Random hexamer primers and RevertAid H Minus Reverse Transcriptase (Thermo Fisher Scientific, Massachusetts, USA) were used in the synthesis of the cDNA. cDNA reaction was performed at the following conditions: 10 min at 25 °C, 30 min at 42 °C and 10 min at 70 °C. All samples were tested in three separate multiplex real-time polymerase chain reaction (real-time PCR) tests using QuantiTect™ Multiplex PCR or NoRox PCR Kit (Qiagen, Hilden, Germany). Primers and probes for seasonal influenza A [25–27] (with influenza A(H3)primer and probe sequences courtesy of Erasmus Medical Centel, Rotterdam, Netherlands) and B viruses [28], respiratory syncytial virus [28], adenovirus [29], rhinovirus [30] and coronavirus (229E, HKU1, NL63 and OC43) [31] (with probe sequences courtesy of P. Simmonds and K. Templeton, personal communication) are previously published. Some modifications have been made in the probe of influenza A(H1)pdm09 [27]. Primer and probe sequences for real-time PCR are available on request. The thermal profile for all three multiplex were 95 °C for 15 min for enzyme activation followed by 50 cycles at 95 °C, 55 °C and 45 °C, 45 s in each step using Stratagene Mx3005P thermal cycler. The respiratory viruses selected for this study represent the virus panel that we use for our standard respiratory virus surveillance.

## Results

Altogether, 90 surface samples and four air samples were collected during weeks 5–7/2016 (Table 1). Nucleic acid of at least one respiratory virus was detected in 9 surface samples (10%). Of surface samples from week 5, 6 and 7, two of 25 (8%), three of 31 (9.7%), and four of 34 (11.8%) respectively were positive.

Viral nucleic acid was found in samples from the surfaces of a plastic toy dog in the children’s playground (two of three swabs, 66.7%), hand-carried luggage trays at the security check area (four of eight, 50%), the buttons of the payment terminal at the pharmacy (one of two, 50%), the handrails of stairs (one of seven, 14%) and the passenger side of desk and divider glass at the passport control points (one of three, 33.3%).

Both rhinovirus and coronavirus OC43 were detected in the same sample from buttons of the payment terminal at the pharmacy. The samples from the armrest of chairs at the waiting area (6 samples) and the samples from the handrails of an escalator (10 samples) were negative. One sample (of 7) from stair handrails was positive for coronavirus OC43. None of the samples from toilets (upper surface the toilet bowl lid, button for flushing, and door lock; 14 samples from different toilets for each surface type) were positive for any of the tested respiratory viruses. No respiratory virus was detected in three samples one from each of the buttons of an elevator, the trolley handles for luggage or the touch screen on the check-in machine.

One of the four air samples (25%) from week 5 between 11:00 h to 11:33 h tested positive for adenovirus.

Among the 10 respiratory virus findings at various sites, in order of frequency these were rhinovirus (four of ten, 40%, from surfaces); coronavirus (three of ten, 30%, from surfaces); adenovirus (two of ten, 20%, 1 surface sample, 1 air sample); and influenza A (one of ten, 10%, surface sample). Subtyping of the influenza A virus by real-time PCR and by sequencing of the hemagglutinin gene was attempted but proved unsuccessful.

The Ct-values of the real time PCR readouts ranged from 36.15 to 41.59.

## Discussion

We performed systematic sampling of frequently touched surfaces in the passenger pathways of a major airport during the seasonal influenza epidemic, and detected respiratory virus nucleic acid in 10 % of the samples. We also took a small number of air samples, 25% of which were positive for respiratory virus nucleic acid. Our finding supports the concept of identifying steps in the passenger process for potential transmission of respiratory viruses, and informs planning for preventive measures to reduce secondary spread. This knowledge helps in the recognition of hot spots for contact transmission risk, which could be important during an emerging pandemic threat or severe epidemic.

Our main findings identify that respiratory virus contamination of frequently touched surfaces is not uncommon at airports; and that plastic security screening trays appear commonly contaminated. The latter is consistent with security procedures being an obligatory step for all departing passengers, and that each security tray is rapidly recycled and potentially touched by several hundred passengers per day. Also, that plastic security trays are non-porous and virus survival is known to be prolonged [32, 33].

In a previous study, environmental sampling for respiratory pathogens in Jeddah airport during the 2013 Hajj season revealed presence of viral nucleic acid in 5.5% of air and 17.5% of surface specimens, most commonly from chair handles [22]. The viral pathogens detected in that study included influenza B virus, human adenovirus, and human coronavirus OC43/HKU1. In a different context, a study on virus shedding from patients and environmental deposition of influenza A(H1N1)pdm09 virus, 4.9% of the swabs from surfaces in the immediate vicinity of the patient were positive for viral nucleic acid, and of the samples cultured, 11.7% were positive [17]. Viral nucleic acid was also detected in air samples collected around five of 12 (42%) patients.

The presence of viral RNA of pathogens frequently circulating in the community during the sampling period is not unexpected, as many viruses survive on surfaces for extended periods [32, 34] and viral nucleic acid can be detected for longer than the time for which viability and transmissibility may persist [35]. Influenza A virus has been reported to survive for 24–48 h on non-porous and up to 8–12 h on porous surfaces [32, 33]. For human rhinoviruses, survival times of infective virus and viral RNA have been reported as > 24 h and > 48 h, respectively [20]. Results for survival times for coronavirus on surfaces vary; one investigation found SARS could not be recovered from dried paper, suggesting its survival time was limited [36]. However, findings from other studies indicate survival times for SARS and Middle East respiratory syndrome coronavirus (MERS-CoV) can be much longer, depending on the surface [35]. In a hospital setting in Taiwan, where there was a significant outbreak of SARS, PCR results indicated the presence of SARS on a variety of surfaces suggesting surface contamination should be considered a risk; however no viable virus was cultured [37]. Similarly, in Toronto surface samples in a hospital were positive by PCR for SARS [38]. MERS-CoV has been shown to remain viable on surfaces for longer than influenza A(H1N1) virus [39].

We used a PCR panel employed in our standard respiratory virus surveillance to detect viral nucleic acid in the samples. We did not attempt to recover live viruses by cell culture. Although PCR methodology has limitations because it does not demonstrate the presence of infective virus, it is commonly used to detect the presence of a virus. Also limiting is that the total number of samples taken is relatively small (*n* = 94). Our sample collection took place within three hours of the daily traffic peaks, well within the reported survival times on surfaces associated with common respiratory viruses. However, whilst the Ct values in our study are similar to those for surface samples in other studies, e.g. [17], these are relatively high, suggesting a low viral load on the surfaces that tested positive, and possibly not constituting the minimum infective dose. Likely due to the high Ct value, subtyping for the influenza A positive specimen was not successful and did not provide information on the origin of the viral strain and its epidemiological context. Alternatively sampling and recovery techniques may have been relatively inefficient, giving an illustration of the potential for transmission, but underestimating the true transmission potential of contaminated surfaces and air. Data concerning the infectious dose specifically for indirect contact are lacking [17]. Killingley and colleagues used a logical argument to conclude that their level of influenza A surface contamination on its own did not represent an infectious dose [17]. The reasoning was that as the copy count in their surface samples approximately only equated to that needed for aerosol transmission, and the likelihood that higher counts are required for indirect transmission, their surface contamination doses would not have been infective. In this study Ct values were similar to Killingley et al. [17], so likewise it is reasonable to conclude that the environmental contamination we identified may not always (or ever) have constituted an infective dose. However, we are unable to determine precisely when each surface became contaminated, and therefore cannot exclude a higher viral load at an earlier time point. Likewise, we cannot establish the efficiency of our sampling technique and the readouts we have may be low due to sampling and recovery techniques. Notwithstanding, we establish the potential for virus transmission from several surfaces. On that basis we do not feel that the potential for transmission can be satisfactorily excluded based on our data.

As previously mentioned, we found the highest frequency of respiratory viruses on plastic trays used in security check areas for depositing hand-carried luggage and personal items. These boxes typically cycle with high frequency to subsequent passengers, and are typically seized with a wide palm surface area and strong grip. Security trays are highly likely to be handled by all embarking passengers at airports; nevertheless the risk of this procedure could be reduced by offering hand sanitization with alcohol handrub before and after security screening, and increasing the frequency of tray disinfection. To our knowledge, security trays are not routinely disinfected. Although this would not eliminate all viruses on hands, (e.g. alcohol gels have been found to be less effective than hand-washing for rhinovirus) [40, 41], it is effective for many viruses, including influenza [42]. In most studies comparing plain soap with alcohol based solutions, the alcohol based solutions were found to be more effective. No respiratory viruses were detected in a considerable number of samples from the surfaces of toilets most commonly touched, which is not unexpected, as passengers may pay particular attention to limiting touch and to hand hygiene, in a washroom environment. Moreover, we did not conduct tests for any enteric viruses.

When an emerging pandemic threat is identified, measures taken to reduce the risk of transmission in an airport, and similar hub environments, could include reducing the risk of indirect transmission, addressing passenger distancing in order to reduce transmission at close proximity (i.e. short range aerosol [43] and droplet transmission), for example in dense queues or at service counters and immigration procedures, enhancing promotion of hand hygiene and respiratory etiquette, and possibly arriving traveler screening procedures. The possible airborne transmission risk can be reduced by ensuring adequate ventilation to dilute pathogen concentrations to sufficiently low levels [44]. Guidelines to mitigate transmission of communicable disease have been issued by Airports Council International [45] and International Civil Aviation Organization [46], but they focus on (exit) screening and handling an individual suspected of having a communicable disease that poses a serious public health risk. A modelling study for entry screening indicated that even in the most optimistic scenarios, the majority of cases of emerging infections would be missed [47]. However, measures preventing transmission locally could be enhanced, for example by improving hand sanitization opportunities where intense, repeat touching of surfaces takes place such as immediately before and after security screening, by enhancing cleaning of frequently touched surfaces, by increased use of non-touch devices, or by effective barriers for face-to-face droplet contact at service counters. Many cleaning agents, household (antibacterial) wipes and anti-viral tissues are able to rapidly render influenza virus nonviable [48], offering multiple simple possibilities and opportunities for reducing the risk of indirect contact transmission.

## Conclusions

Detection of pathogen viral nucleic acids indicates viral surface contamination at multiple sites associated with high touch rates, and suggests a potential risk in standard passenger pathways at airport sites. Security check trays appear to pose the highest potential risk and are used by virtually all embarking passengers; they have the potential to be especially problematic if a severe pathogen with an indirect transmission mechanism were to pose a threat for international spread. Public surface transport has been shown to be associated with acute respiratory infections [49], stressing the need to also investigate the role of various traffic hubs in transmission, including airports, ports and underground stations.

## Abbreviations

Ct value: Cycle threshold value
MERS-CoV: Middle East respiratory syndrome coronavirus
PCR: Polymerase chain reaction
SARS: Severe Acute Respiratory Syndrome
VTM: Viral transport medium
